# The Acute Toxicity and Cardiotoxic Effects of Levofloxacin on Zebrafish (*Danio rerio*)

**DOI:** 10.3390/toxics13020122

**Published:** 2025-02-05

**Authors:** Yixiao Wu, Wenjing Yu, Zhenyan Song, Jiawei He, Ze Li, Qi Chen, Shiwei Wang, Ping Li, Shaowu Cheng

**Affiliations:** 1 School of Integrated Chinese and Western Medicine, Hunan University of Chinese Medicine, Changsha 410208, China; yixiao@stu.hnucm.edu.cn (Y.W.); wjyu@stu.hnucm.edu.cn (W.Y.); songzhenyan2013@hnucm.edu.cn (Z.S.); 20223812@stu.hnucm.edu.cn (J.H.); jylz060@stu.hnucm.edu.cn (Z.L.); chenqi@stu.hnucm.edu.cn (Q.C.); wsw@stu.hnucm.edu.cn (S.W.); lp2025@stu.hnucm.edu.cn (P.L.); 2 Key Laboratory of Hunan Province for Integrated Traditional Chinese and Western Medicine on Prevention and Treatment of Cardio-Cerebral Diseases, Hunan University of Chinese Medicine, Changsha 410208, China; 3 The Second Affiliated Hospital of Hunan University of Traditional Chinese Medicine, Hunan University of Chinese Medicine, Changsha 410208, China; 4 School of Medicine, Hunan University of Chinese Medicine, Changsha 410208, China

**Keywords:** levofloxacin, zebrafish, embryotoxicity, cardiotoxicity, emerging contaminants, gene expression

## Abstract

Emerging contaminants refer to chemical substances that have not been widely regulated but possess the potential to cause adverse effects on both the environment and human health. Antibiotics, as emerging contaminants, pose significant threats to ecosystems and human health due to their widespread use and persistence in the environment. Levofloxacin, a broad-spectrum fluoroquinolone antibiotic, is commonly employed in the treatment of bacterial infections, and has been frequently detected in environmental matrices and freshwater systems. In this study, we assessed the effects of levofloxacin on hatchability, mortality rates, malformations, behavioral changes, and cardiac development in zebrafish embryos by exposing them to varying concentrations of levofloxacin (0, 0.5, 1, 2, 4, and 8 mM). Our results demonstrate that levofloxacin exposure significantly impaired the growth and development of zebrafish larvae, particularly at higher concentrations. Notable effects included reduced body length, abnormal yolk sac and swim bladder development, pericardial edema, prolonged distances between the sinus venosus and arteriolar bulb (SV-BA), and disruptions in heart rate. Quantitative PCR analysis further revealed that levofloxacin exposure significantly upregulated the expression of key cardiac development genes in zebrafish larvae, including *nppa*, *myh6*, *cacna1ab*, *myl7*, *gata4*, *nkx2.5*, *tbx2b*, and *tbx5b*. These findings indicate that levofloxacin exposure exerts significant toxic effects on both embryonic and larval growth as well as heart development and gene expression in zebrafish. This study provides critical insights into the potential ecological risks posed by levofloxacin along with other antibiotics while laying a foundation for further investigation into their toxicological mechanisms.

## 1. Introduction

Emerging contaminants (ECs) are chemicals introduced into the environment through various human activities, such as household use, agriculture, and industrial processes. Advances in analytical technologies have increased the detection and awareness of these compounds [1]. ECs comprise a diverse array of exogenous substances, including pharmaceuticals and personal care products (PPCPs), per- and polyfluoroalkyl substances (PFAS), pesticides, micro/nanoplastics, and antibiotic resistance genes (ARGs) [2]. Among these, antibiotics, heavy metals such as lead, pesticides, persistent organic pollutants, and wastes containing emerging contaminants have become focal points for international organizations in the management of synthetic compounds [3].

For example, in 2010, approximately 63,200 metric tons of antibiotics were used in animal agriculture, primarily as growth promoters in poultry production and agriculture and aquaculture [4]. However, about 95% of antibiotics administered to animals are excreted unmetabolized or as metabolites, and often enter aquatic systems through agricultural runoff when used as fertilizers [5]. Antibiotics’ prevalence and their role in promoting bacterial resistance have made them a key focus of environmental research.

Fluoroquinolones (FQs), including ciprofloxacin, levofloxacin, and moxifloxacin, are broad-spectrum antibiotics extensively utilized across human medicine, veterinary practice, and household settings. As a result, they represent one of the most frequently prescribed classes of antibiotics [6]. Due to their poor biodegradability, prolonged or excessive use of FQs has resulted in environmental contamination, particularly in aquatic systems [7]. Levofloxacin (LEV), a third-generation FQ with broad-spectrum activity, has been found to contribute to low-level antimicrobial resistance in aquatic bacteria exposed over time, increasing the risk of “superbug” formation through continuous mutation [8]. Recent reports have detected levofloxacin in various river systems, with concentrations reaching 3600 ng/L in Japan and 6800 ng/L in China, 34–438 ng/L in Portugal, and 2030 ng/L in Indian wastewater in 2019 [9,10]. Even at trace concentrations (ranging from ng/L to µg/L), antibiotics may promote the development of resistant bacterial strains, posing a threat to both human health and ecological systems [11,12].

The zebrafish (*Danio rerio*) has emerged as a prominent model organism in toxicological research due to its high reproductive capacity, small size, low cost, ease of maintenance, and the transparency of its embryos, which facilitates direct observation. Zebrafish have been widely used for assessing drug side effects, evaluating pesticides, and conducting environmental toxicity studies [13,14]. Chemical pollutants have been demonstrated to impact multiple facets of fish embryonic development, including growth, neurodevelopment, and cardiovascular function, with the heart playing a pivotal role in oxygen circulation and distribution throughout the organism [15]. As such, cardiac development damage has been considered an important indicator in the evaluation of pollutant toxicity. Additionally, zebrafish possess a well-characterized genetic background, with approximately 70% homology to human genes, and 84% of human disease-related genes have corresponding counterparts in the zebrafish genome [16]. Therefore, zebrafish are widely used for research on disease mechanisms, drug screening, and toxicological assessments [17,18]. While previous studies have investigated the effects of fluoroquinolones on oxidative stress, neurobehavior, and embryonic heart development in zebrafish [19,20,21], research focusing on levofloxacin’s specific developmental toxicity remains limited.

In summary, the monitoring of emerging environmental contaminants, particularly levofloxacin, has attracted significant attention, though toxicological research in this field remains in its early stages. This study aims to investigate the effects of levofloxacin exposure on zebrafish embryo development using the wild-type AB line of zebrafish. By analyzing hatchability, mortality, malformations, behavioral changes, and cardiac toxicity, this research provides novel insights into the ecological toxicity of levofloxacin through a combination of morphological, behavioral, and molecular biological approaches.

## 2. Materials and Methods

### 2.1. Reagents and Instruments

We obtained the following: levofloxacin (CAS: 100986-85-4, purity > 99%, MedChemexpress, Monmouth Junction, NJ, USA); TRIzol reagent (15596018, Thermo Fisher Scientific, Waltham, MA, USA); Reverse Transcription Kit (0521751, Suzhou, Nearshore Protein Science and Technology Co., Hangzhou, China) Magic SYBR Mixture (CW3008M, Kangwei Century, Beijing, China); embryo culture medium (Shanghai Feixi Biotechnology Co., Ltd., Shanghai, China); and 3-ethoxyphenylmethanesulfonate (CAS: 886-86-2, Aladdin, Shanghai, China).

We also obtained the following: a biological microscope (MOTIC Moticam Pro 205A, Chai Wan, Hong Kong); a stereomicroscope (Leica S9i, Wetzlar, Germany); a Sorvall™ Legend™ Micro 17R Microcentrifuge (Thermo Fisher, Waltham, MA, USA); a Cytation3 Multi-Mode Microplate Reader (Bio-Tek, Winooski, VT, USA); a T100™ Thermal Cycler Real-Time Fluorescence Quantitative PCR Amplifier; and a Zebrafish Heart Rate and Behavioral Analysis System (ViewPoint ZebraLab, Lyon, France).

### 2.2. Zebrafish Rearing and Embryo Collection

The wild-type AB line of zebrafish was purchased from Hubei Chuangxin Biotechnology Co., Ltd. (Jingzhou, China) and maintained in a breeding system at the Hunan Provincial Key Laboratory of Hunan University of Traditional Chinese Medicine (Changsha, China). After quarantine, the zebrafish were acclimated under the following controlled conditions: pH (6.8–8.5), temperature (24–28 °C), alkalinity (50–100 mg/L), salinity (0.5–2.0 g/L), and dissolved oxygen (over 4 mg/L), with a light/dark cycle of 14 h light/10 h dark (light period from 8 a.m. to 9 p.m.). The fish were fed live artemia three times a day. Two males and one female zebrafish were placed in a breeding tank the night before the experiment, kept in the darkness overnight, and the following morning, after removing the divider, spawning was induced under light conditions. Embryos were collected 1–2 h post-spawning and placed in dishes containing embryo culture medium. These dishes were then transferred to an incubator at 28 °C for hatching. At 6 h post-fertilization (hpf), embryos with normal development were randomly selected for subsequent drug exposure experiments.

### 2.3. Levofloxacin Exposure

Levofloxacin was dissolved in embryo culture medium to create six concentration gradients for exposure: 0, 0.5, 1, 2, 4, and 8 mM. Three replicates were prepared for each concentration, with 30 embryos per replicate. At 6 hpf, embryos with normal development and transparency, and at the same developmental stage, were selected under a microscope and placed in 6-well plates. A total of 5 mL of exposure solution was added to each well, and the embryos were incubated in a constant-temperature incubator until 96 hpf or 120 hpf. The exposure solution was refreshed every 24 h with freshly prepared solutions, with concentrations selected based on the literature and preliminary experimental results [20]. The hatchability and survival rates of zebrafish embryos, as well as the teratogenic and lethal effects on the larvae, were observed and recorded at least once every 24 h. Phenotypic images of live embryos exposed to levofloxacin were captured using a microscope, with imaging settings remaining consistent throughout the experiment.

### 2.4. Observation and Measurement of Zebrafish Developmental Parameters

#### 2.4.1. Morphological Observation

Starting at 24 hpf, the embryos were observed daily using a stereomicroscope to assess desiccation and mortality, including the death of embryos or post-desiccation larvae. Death was determined based on the observation of embryo coagulation, a cessation of heartbeat, or a lack of movement. The number of dead embryos was recorded, and the dead embryos were immediately removed to calculate the survival rate. The hatchability rate was calculated at 72 hpf. At 96 hpf and 120 hpf, the larvae were anesthetized with 3-ethoxyphenylmethanesulfonate and subsequently transferred to a glass slide using a pipette. The developmental progression of the larvae was monitored and photographed under a biological microscope. Key morphological parameters, including body length, SV-BA length, and the areas of structures such as the pericardium, the yolk sac, and the swim bladder, were quantified using ImageJ-win64software.

#### 2.4.2. Heart Rate Measurement

After 72 h of levofloxacin exposure, the larvae were transferred onto a glass slide using a pipette and placed under a microscope. High-speed imaging was performed using the MicroZebraLab zebrafish heart rate and blood flow analysis software to capture and analyze alterations in heart rate. Three larvae from each concentration group were randomly selected for heart rate monitoring. The number of heartbeats within 20 s was recorded for each larva, and the observation was repeated three times. Before observation, the larvae were allowed to acclimate under the microscope for 1 min.

#### 2.4.3. Assessment of Locomotor Behavior

Four days after levofloxacin exposure, eight larvae from each experimental group were randomly selected for behavioral analysis. The selected larvae were carefully placed into a 48-well plate, with one larva and 1 mL of embryo culture medium in each well. The 48-well plate was then placed in a behavior analysis system. A high-throughput behavior tracking analyzer (ViewPoint ZebraLab) was employed to monitor and record the behavioral trajectories of the zebrafish larvae, allowing for the assessment of levofloxacin’s impact on their motor function. The analysis procedure included a 10 min light adaptation period, followed by three light/dark cycles (10 min of darkness, then 10 min of light) [22]. Finally, the total movement distance, low-to-medium speed movement distance (1–12 cm/s), and high-speed movement distance (>12 cm/s) of the larvae in each concentration group were statistically analyzed.

### 2.5. RNA Extraction and Quantitative Real-Time PCR Analysis

Zebrafish larvae (120 hpf) from each concentration group were collected, immersed in ice-cold water until fully anesthetized, and then dried to remove excess water. Total RNA was extracted from the larval samples using TRIzol reagent, and the RNA concentration was determined. The extracted RNA was stored at −80 °C. cDNA synthesis was performed using the NovoScript^®^ mRNA First Strand cDNA Synthesis Kit. In a 40 μL reaction system, 2000 ng of RNA was used for first-strand cDNA synthesis under the specified temperature and time conditions. The synthesized cDNA was stored at −20 °C. Primers were designed using Primer Premier 6.0 software, with melting temperatures (Tm) set at 55 °C ± 5 °C and amplicon sizes ranging from 100 to 300 bp (Table 1). All primers were validated before use. Quantitative PCR was performed using SYBR Green dye to measure the mRNA expression levels of target genes, with β-actin as an internal control. Each sample was analyzed in triplicate, and the data were compared with the control group. The relative mRNA expression levels of the target genes were calculated using the 2^−ΔΔCq^ method [23].

### 2.6. Statistical Analysis

The quantification of zebrafish larval body length, SV-BA length, swim bladder area, and yolk sac area was performed using ImageJ software. Statistical analysis was conducted using SPSS 25.0 and GraphPad 8.0.1. The data are presented as the mean ± standard error of the mean (SEM). One-way analysis of variance (ANOVA) was used for comparisons between groups, with statistical significance defined as *p* < 0.05.

## 3. Results

### 3.1. Developmental Toxicity of Levofloxacin Exposure in Zebrafish Embryos

**Figure 1:**
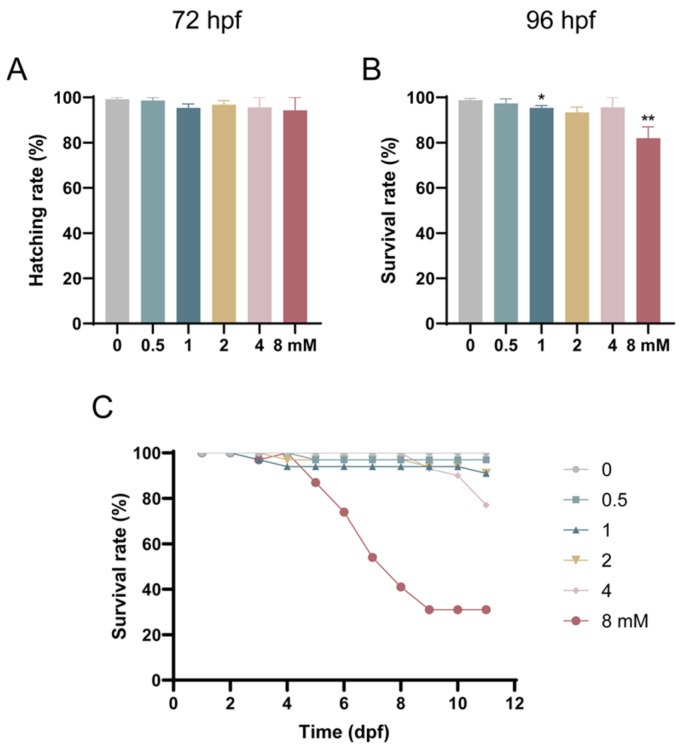
Developmental toxicity of levofloxacin exposure in zebrafish embryos. (**A**) Hatching rates of zebrafish embryos exposed to levofloxacin at 72 hpf; (**B**) survival rates of zebrafish embryos exposed to levofloxacin at 96 hpf; (**C**) survival rates of zebrafish larvae at different time points under different concentrations of levofloxacin. * *p* < 0.05; ** *p* < 0.01 vs. 0 mM.

To investigate the developmental toxicity of levofloxacin exposure in zebrafish embryos, the embryos were exposed to different concentrations of levofloxacin, and hatch rates were calculated for each group. At 72 hpf, no significant changes in hatch rates were observed in all exposure groups compared to the control (0 mM group), with hatch rates exceeding 94% (Figure 1A). However, at 96 hpf, survival rates were decreased in the exposed groups compared to the control, with the 1 mM and 8 mM groups showing a significant decrease in survival (*p* < 0.05) (Figure 1B). Overall, levofloxacin exposure negatively affected the early development of zebrafish embryos, leading to delayed hatching and reduced survival rates. Figure 1C illustrates the survival rates of zebrafish larvae at various time points under different concentrations of levofloxacin. At 5 days post-fertilization (dpf), zebrafish larvae from the experimental groups were transferred to normal culture water. Between 5 and 9 dpf, the survival rate of the 8 mM group gradually decreased, while the survival rates of the other groups remained relatively stable. After 9 dpf, a decline in survival rate was observed in the 4 mM group. Although no significant differences were observed among the 0.5, 1, 2, and 4 mM groups, the cumulative survival rate in all exposure groups remained lower than that of the control group. These results indicate that the toxic effects of levofloxacin extend beyond the exposure period, as zebrafish larvae continued to exhibit irreversible adverse effects even after being transferred to normal water for further development.

### 3.2. Effects of Levofloxacin Exposure on Zebrafish Larval Morphology

#### 3.2.1. Body Length

**Figure 2:**
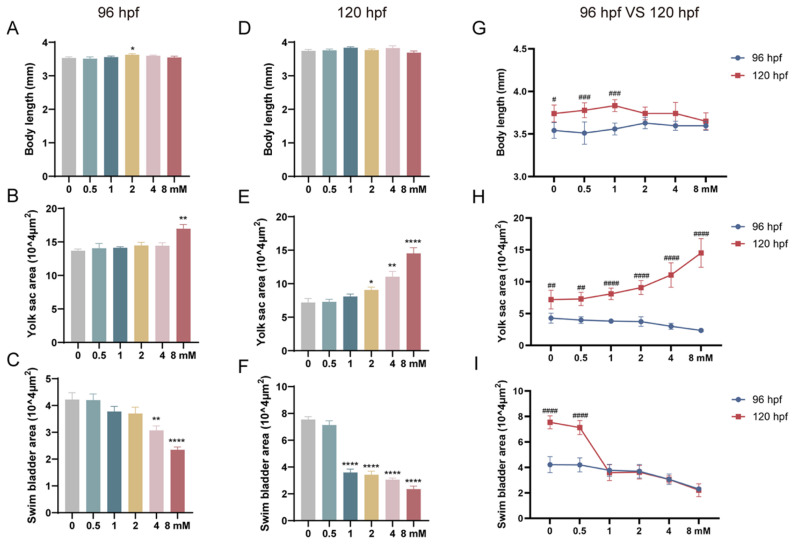
Morphological effects of levofloxacin exposure on zebrafish larvae at 96 hpf and 120 hpf. (**A**) Body length at 96 hpf; (**B**) yolk sac area at 96 hpf; (**C**) swim bladder area at 96 hpf; (**D**) body length at 120 hpf; (**E**) yolk sac area at 120 hpf; (**F**) swim bladder area at 120 hpf. * *p* < 0.05; ** *p* < 0.01; **** *p* < 0.0001 vs. 0 mM. (**G**) Comparison of body length between 96 hpf and 120 hpf; (**H**) comparison of yolk sac area between 96 hpf and 120 hpf; (**I**) comparison of swim bladder area between 96 hpf and 120 hpf. # *p* < 0.05; ## *p* < 0.01; ### *p* < 0.001; #### *p* < 0.0001 vs. same concentration at 96 hpf.

The morphological development of zebrafish larvae exposed to different concentrations of levofloxacin was observed and compared to the 0 mM control group. At both 96 hpf and 120 hpf, no significant differences in body length were observed among the exposure groups (Figure 2A,D). However, larvae exposed to 2, 4, and 8 mM levofloxacin showed a significant delay in body length development between 96 hpf and 120 hpf (Figure 2G).

#### 3.2.2. Yolk Sac

**Figure 3:**
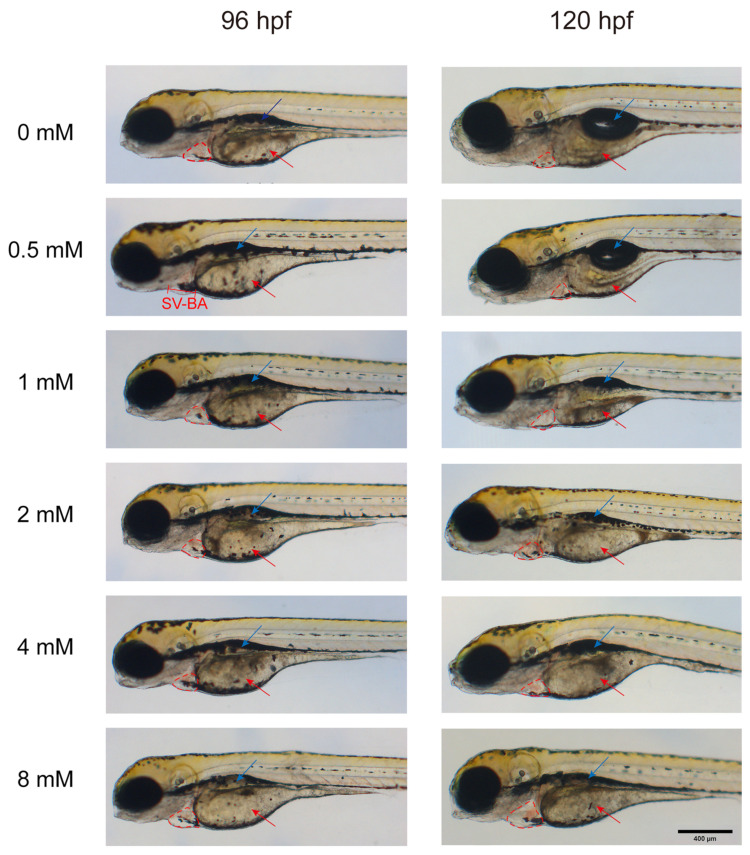
Morphological effects of levofloxacin exposure on zebrafish larvae. The red arrow indicates the yolk sac, the blue arrow indicates the swim bladder, and the red dashed line highlights the pericardial area. SV-BA: Length of sinus venosus to bulbus arteriosus.

To evaluate the impact of levofloxacin exposure on yolk sac development, the zebrafish were anesthetized and fixed, and the yolk sac area was quantified under a microscope. As depicted in Figure 3, the red arrows indicate the location of the yolk sac. At 96 hpf, only the 8 mM group showed a significant increase in yolk sac area compared to the control group (*p* < 0.01) (Figure 2B). At 120 hpf, the 4 mM and 8 mM exposure groups showed a significant increase in yolk sac area (*p* < 0.0001), while the 2 mM group also showed a significant increase (*p* < 0.05). Additionally, although the yolk sac area in the 0.5 mM and 1 mM groups increased with increasing concentrations of levofloxacin, no statistically significant differences were detected (Figure 2E). A comparison between 96 hpf and 120 hpf revealed that the severity of yolk sac mal-formations also increased with increasing exposure concentration (Figure 2H).

#### 3.2.3. Swim Bladder

The swim bladder, indicated by the blue arrows in Figure 3, was examined in zebrafish larvae exposed to different concentrations of levofloxacin. At 96 hpf, the swim bladder area was significantly smaller in the 4 mM (*p* < 0.01) and 8 mM (*p* < 0.0001) groups than in the control group, with other experimental groups showing a similar trend of reduction (Figure 2C). At 120 hpf, all experimental groups, except for the 0.5 mM group, exhibited significantly smaller swim bladder areas compared to the control group (*p* < 0.0001) (Figure 2F). A concentration-dependent decrease in swim bladder area was observed at both 96 hpf and 120 hpf, reflecting impaired swim bladder expansion in zebrafish larvae exposed to higher concentrations of levofloxacin. In the 0 and 0.5 mM groups, the swim bladder area at 120 hpf was significantly larger than at 96 hpf (*p* < 0.0001), whereas no significant difference was observed between 96 hpf and 120 hpf in the 1, 2, 4, and 8 mM groups (Figure 2I).

### 3.3. Levofloxacin Exposure Induces Cardiotoxicity in Zebrafish Larvae

**Figure 4:**
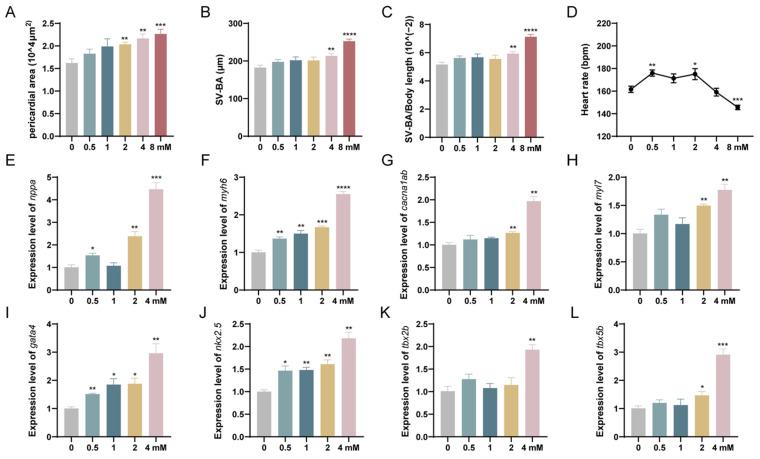
Cardiotoxic effects of levofloxacin exposure on zebrafish larvae. (**A**) Pericardial area of zebrafish larvae; (**B**) SV-BA length of zebrafish larvae; (**C**) ratio of SV-BA to body length of zebrafish larvae; (**D**) heart rate (beats per minute) of zebrafish larvae after 72 h of levofloxacin exposure; (**E**–**H**) expression of cardiac development marker genes (*nppa*, *myh6*, *myl7*, *cacna1ab*); (**I**–**L**) expression levels of major transcription factors (*gata4*, *nkx2.5*, *tbx2b*, *tbx5b*). * *p* < 0.05; ** *p* < 0.01; *** *p* < 0.001; **** *p* < 0.0001 vs. 0 mM.

The effects of levofloxacin exposure on the cardiovascular system of zebrafish larvae were observed under a microscope. At 96 hpf, the area of the pericardial region in zebrafish larvae exposed to 2 mM (*p* < 0.01) and 8 mM (*p* < 0.001) levofloxacin was significantly larger compared to the 0 mM control group, while no significant changes were observed in the 0.5 mM and 1 mM groups, although a general increasing trend was observed (Figure 4A). Similarly, at 96 hpf, the length of the venous sinus-arterial bulb (SV-BA) and the ratio of SV-BA to body length were significantly increased in the 4 mM (*p* < 0.01) and 8 mM (*p* < 0.0001) groups (Figure 4B,C). Figure 4D shows the results of heart rate measurements taken at 72 h after exposure. Compared to the 0 mM group, the heart rate in the 0.5 mM and 2 mM groups was significantly higher (*p* < 0.05), while the heart rate in the 8 mM group was significantly decreased (*p* < 0.0001). To further confirm the cardiotoxic effects of levofloxacin, we performed cardiac gene expression analysis on zebrafish larvae exposed to levofloxacin. Real-time quantitative PCR (qPCR) revealed that the expression levels of key cardiac development marker genes (*nppa*, *myh6*, *myl7*, *cacna1ab*) and major transcription factors (*gata4*, *nkx2.5*, *tbx2b*, *tbx5b*) were significantly upregulated in the exposure groups compared with the 0 mM group in a concentration-dependent manner (Figure 4E–L).

### 3.4. Effects of Levofloxacin Exposure on Zebrafish Larval Locomotor Behavior

**Figure 5:**
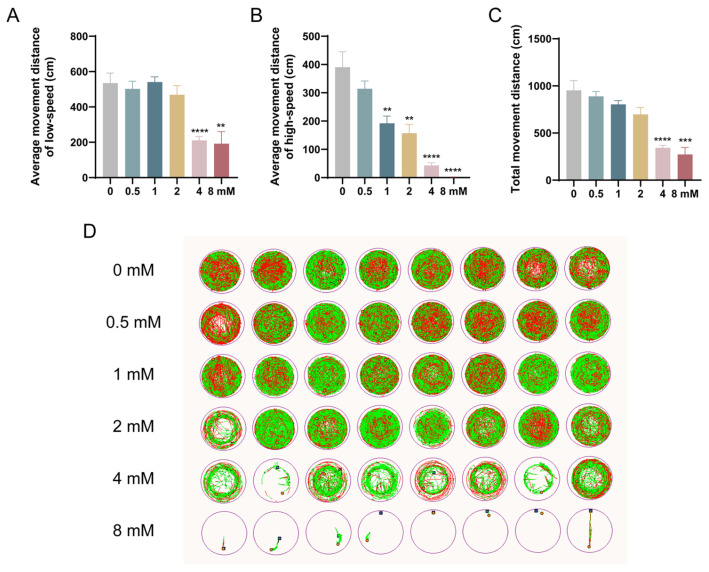
Effects of 96 h levofloxacin exposure on zebrafish larval swimming behavior. (**A**) Low-to-moderate speed movement distance of zebrafish larvae in each concentration group; (**B**) high-speed movement distance of zebrafish larvae in each concentration group; (**C**) total movement distance of zebrafish larvae in each concentration group; (**D**) swimming trajectories of zebrafish larvae in each concentration group. Green trajectories represent low-to-moderate speed movement (1–12 cm/s), and red trajectories represent high-speed movement (>12 cm/s). ** *p* < 0.01; *** *p* < 0.001; **** *p* < 0.0001 vs. 0 mM.

The behavioral changes in zebrafish larvae at various concentrations after 96 h of exposure to levofloxacin are presented in Figure 5. To assess motor activity, the distance traveled at different speeds was measured. In the statistics of low-to-medium speed movement distance, the 0.5 mM, 1 mM, and 2 mM groups did not show significant changes compared with the 0 mM group, whereas the low-to-medium speed movement distance was significantly lower in the 4 mM (*p* < 0.0001) and 8 mM (*p* < 0.01) groups (Figure 5A). However, the 4 mM (*p* < 0.0001) and 8 mM (*p* < 0.01) exposure groups exhibited a significant reduction in low-to-medium speed movement distance. In terms of high-speed movement and total distance traveled, both parameters decreased with increasing exposure concentrations, showing a dose-dependent effect (Figure 5B,C). Specifically, the high-speed movement distance was significantly reduced in the 1 mM and 2 mM groups (*p* < 0.01), as well as in the 4 mM and 8 mM groups (*p* < 0.0001) (Figure 5B). Similarly, the total movement distance was significantly reduced in the 4 mM (*p* < 0.0001) and 8 mM (*p* < 0.001) groups compared to the 0 mM control group (Figure 5C).

## 4. Discussion

The purpose of this study was to evaluate the toxic effects of levofloxacin on the embryonic development of zebrafish. As a broad-spectrum antibiotic, levofloxacin is widely used in the treatment of bacterial infections in humans and animals. However, due to its limited metabolic degradation in vivo, levofloxacin is released into the environment and accumulates in aquatic and terrestrial ecosystems, contributing to the development of antimicrobial resistance. It has thus become a common contaminant in aquatic environments [24,25]. While the potential effects of levofloxacin on aquatic organisms, particularly zebrafish embryos, have received some attention, systematic studies of its developmental toxicity remain scarce. This research paper provides a detailed analysis of the dose-dependent toxic effects of levofloxacin on zebrafish embryonic development, focusing on growth and cardiac developmental toxicity. These findings not only provide scientific evidence for evaluating the ecological toxicity of levofloxacin, but also open new avenues for further investigation into the underlying toxicological mechanisms.

The findings of this study underscore the significant toxic effects of levofloxacin on zebrafish embryonic development. Previous research has suggested that reduced hatchability may be linked to mitotic arrest, disrupted embryogenesis, and abnormal embryonic structures and functions [26]. In this study, although the direct effects of levofloxacin on the hatchability of zebrafish embryos were not significant at different concentrations, the hatching process was delayed with increasing concentrations, and the survival rate of embryos significantly decreased. This suggests that higher concentrations exacerbate the toxic effects, resulting in more pronounced hatching delays and mortality. Body length, an important parameter reflecting embryonic growth, was also reduced, indicating suppressed larval growth. Although no significant trends were observed between the different concentration groups at 96 hpf and 120 hpf, larval growth was notably suppressed in the 2, 4, and 8 mM exposure groups compared to the lower concentration groups. This suggests that levofloxacin exposure delays or inhibits the growth of zebrafish larvae, with the potential for more severe developmental impairment under prolonged exposure.

Furthermore, zebrafish larvae exposed to levofloxacin displayed a variety of developmental abnormalities, including yolk sac edema, underinflation of the swim bladder, cardiac hypoplasia, and impaired locomotion. Previous studies have demonstrated that quinolones, including levofloxacin, induce dose-dependent embryonic lethality or severe malformations in zebrafish, such as pericardial edema, cardiac defects, yolk sac edema, shortened body axis, and scoliosis [20]. Phenotypic characterization is a robust indicator widely used in zebrafish embryonic and larval toxicity assessment, and is suitable for high-throughput screening [27]. During zebrafish development, the yolk sac serves as a critical nutrient source, especially during the embryonic and early larval stages, providing energy and essential precursors for proteins such as lipoproteins [28]. Before 6 dpf, when larvae are not capable of independent feeding, the yolk sac is its primary source of energy and nutrients. Our study found that with increasing concentrations of levofloxacin, the extent of yolk sac malformation intensified, suggesting that levofloxacin exposure interferes with yolk sac absorption and causes swelling.

Swim bladder development, a tightly regulated process, plays essential roles in buoyancy regulation, sound sensing, and aiding respiration [29]. It is a critical developmental event in fish species, as it reduces the larval body mass-to-volume ratio, enabling swimming and surface feeding [30]. It has been reported that zebrafish larvae complete swim bladder inflation by 120 hpf [31]. Therefore, in this study, the exposure duration was extended from 96 hpf to 120 hpf to better observe swim bladder developmental abnormalities. Our results indicate that levofloxacin exposure significantly impaired swim bladder development and inflation capacity in zebrafish larvae. At both 96 hpf and 120 hpf, the swim bladder area decreased in all concentration groups in a dose-dependent manner. In the low-concentration groups (0 and 0.5 mM), the swim bladder area significantly increased by 120 hpf compared to 96 hpf, suggesting delayed development with partial recovery. However, in the high-concentration groups, no significant improvement was observed, indicating irreversible damage to swim bladder function. Similar results have been reported in studies involving other contaminants, such as heavy metals and natural pyrethroids, which disrupt normal swim bladder development and cause inflation abnormalities [32,33,34].

The heart is the first organ to form and begin functioning during vertebrate embryonic development. Cardiac circulation is a pivotal event in heart development, with the early embryonic heart initially forming as a linear tubular pump. As development progresses, this tubular heart undergoes twisting to form a spiral structure, a process referred to as cardiac looping. The formation of the ventricle and atrium is critical for the normal delivery of blood to the entire body, and any developmental disruption of these structures often results in an abnormal expression of associated genes [35,36]. Our findings indicate that exposure to levofloxacin significantly increased the size of the zebrafish heart, including the SV-BA distance, the ratio of SV-BA to body length, and the pericardial area. Early studies have also demonstrated that levofloxacin exposure leads to a dose-dependent increase in pericardial area and SV-BA distance [20]. Similar results have been reported, where these changes are considered classic phenotypic markers of cardiac malformations and the most sensitive terminal effects of contaminant exposure [37]. Heart rate, another crucial parameter of cardiac toxicity, reflects potential cardiac dysfunction. Consistent with our results, levofloxacin exposure led to dose-dependent changes in heart rate, with tachycardia observed at low concentrations (0.5–2 mM) and bradycardia at high concentrations (8 mM). This pattern may reflect compensatory mechanisms at lower doses and decompensation due to excessive toxicity at higher doses [20].

During cardiac development, genes such as *nppa*, *myh6*, *myl7*, *cacna1ab*, *gata4*, *nkx2.5*, *tbx2b*, and *tbx5b* play critical roles. Exposure to levofloxacin significantly disrupted the expression of these genes, leading to cardiac malformations and dysfunction. The *nppa* gene, associated with atrial natriuretic peptide expression, is highly expressed during embryonic stages and is upregulated under cardiac stress [38]. The increased *nppa* expression observed in this study aligns with its role in abnormal heart morphogenesis under stress conditions. The existing literature suggests that carboxyl groups at position C1 of the fluorene molecule enhance its acute toxicity to zebrafish embryos, disrupting heart development and affecting heart rate, with abnormal heart morphogenesis associated with the upregulation of *nppa* expression [39]. The *myh6* gene encodes the myosin-heavy chain in cardiac muscle, serving as a marker for cardiac development and participating in the activation of ventricular cardiomyocyte formation [40]. The *myh6* −/− mutant causes the inability of atrial cardiomyocytes to contract and triggers ventricular enlargement due to atrial hypoplasia [41]. Abnormal expression of *myh6* may result in structural and functional defects in the myocardium. Furthermore, *myl7* is a marker for heart development and differentiation, essential for the development and contractility of the heart [42]. The influx of calcium ions is critical for cardiomyocyte contraction, and *cacna1ab*, which encodes a calcium channel, plays a direct role in this process, regulating calcium ion-dependent cellular behaviors [43]. In studies examining the mechanisms of cardiac developmental toxicity, the expression of genes like *myl7* and *cacna1ab* has also been evaluated [37], with results similar to ours being found, where both genes were significantly upregulated. *Gata4*, a transcription factor essential for heart morphogenesis, was significantly upregulated in exposed groups, consistent with its role in heart tube formation and its dysregulation under toxic stress [44]. The *nkx2.5* gene is a key pathogenic gene associated with congenital heart defects and is likely involved in the early steps of heart development. The expression of *nkx2.5* has been detected in differentiating cardiomyocytes in all vertebrate species [35]. Studies have shown that *nkx2.5* plays distinct roles in the development of the ventricle and atrium, with effects on cell number and morphology that vary at different developmental stages [45]. In a study investigating the toxicity of fungicides on zebrafish embryonic hearts [46], abnormal expression of key cardiac regulators such as *gata4* and *nkx2.5* was observed, with *gata4* expression gradually increasing with exposure concentration, consistent with our findings. However, while fungicide exposure was associated with a reduction in *nkx2.5* expression, our study observed a significant upregulation of *nkx2.5* expression under levofloxacin exposure. We hypothesize that species-specific differences may account for the divergent responses to environmental pollutants. Certain pollutants might increase *nkx2.5* expression by activating particular signaling pathways, while others could inhibit its expression by inducing oxidative stress or disrupting DNA repair mechanisms. Nonetheless, it is evident that *nkx2.5* is a crucial transcription factor in cardiac development, with its role in the early stages of heart formation being indispensable. Various environmental pollutants may thus alter the expression patterns of *nkx2.5*, potentially interfering with normal cardiac development. The *tbx2b* and *tbx5b* genes, both part of the T-box transcription factor family, are vital for the development of the atrioventricular canal and overall heart function [47]. Disrupted expression of these genes in this study is consistent with reports of heart defects induced by environmental pollutants [48]. In summary, exposure to levofloxacin significantly disrupted the early expression of genes related to cardiac development, resulting in cardiac malformations and dysfunction, as well as a cascade of severe developmental toxicities in zebrafish hearts.

While previous studies have documented the teratogenic effects and cardiac toxicity of levofloxacin on zebrafish embryos, the impact of this antibiotic on behavior remains unexplored. Behavioral analysis can measure several variables, such as spontaneous movement starting at 17 hpf, tail curling induced by touch starting at 21 hpf, and swimming activity beginning at 27 hpf [49]. These methods typically involve recording the movement of embryos or larvae over a specific period, quantifying their activity, and comparing differences in movement patterns with control groups to reveal distinct phenotypic changes [50]. Zebrafish exhibit strong social behaviors, forming groups with hierarchical structures and showing preferences for conspecifics. They also demonstrate novel responses to new environments or situations, with preferences for factors such as depth, color, and darkness. Both males and females display aggression, fear-like responses, anxiety behaviors, and specific courtship behaviors [51,52]. Similar behaviors were observed in our study. When exposed to alternating light and dark conditions, zebrafish larvae exhibited significantly increased activity, particularly in the dark. The results from behavioral tests indicated that with increasing concentrations of levofloxacin exposure, the swimming activity of zebrafish larvae was notably suppressed, as evidenced by decreased swimming speed and distance. In the low-speed and medium-speed movement categories, there was no significant difference in movement distance between the exposure groups (0.5, 1, 2 mM) and the control group. However, in the high-speed movement category, the distance traveled by the 1 mM and 2 mM exposure groups was significantly reduced compared to the control group. Our experimental setup was consistent with previous studies, where zebrafish were alternated between light and dark environments for 10 min each, repeated three times. The results were similarly consistent, showing that environmental pollutant exposure significantly reduces swimming distance and activity in zebrafish larvae [22]. Previous research has also reported changes in zebrafish behavior due to antibiotic exposure. In contrast to our findings, one study found that exposure to ciprofloxacin increased the swimming distance of adult zebrafish [53]. This discrepancy may be attributed to different impacts of pollutants on the zebrafish nervous and muscular systems. Zebrafish larvae, with their nervous systems and motor neurons still under development, are more vulnerable to neurotoxic pollutants that may disrupt neural signaling or damage neurons, leading to impaired motor control and coordination, which subsequently reduces swimming distance. In contrast, adult zebrafish, with a more mature nervous system, may respond differently to the same toxin. Adult fish possess more robust detoxification and metabolic capabilities, potentially displaying a transient increase in activity in response to low concentrations of pollutants. This may represent a stress response, where the increased activity is an attempt to avoid the perceived “threat,” often referred to as “excitation toxicity.” In addition, the literature has explored the effects of antibiotic exposure on zebrafish learning, memory, and aggression. Our study, however, has not been comprehensive in this regard. Future work will involve continued behavioral assessments in adult zebrafish to further investigate the impacts of levofloxacin on learning, memory, and social behaviors. This highlights the ecological significance of behavioral changes in zebrafish, as such changes can directly influence their adaptability, reproductive success, and even the survival of the species. Consequently, assessing the impact of environmental contaminants—particularly on the behavioral responses of aquatic organisms like fish—is of paramount ecological importance. Furthermore, these behavioral changes suggest that levofloxacin may exert neurotoxic effects during early embryonic development in zebrafish, which warrants further investigation of its influence on neurodevelopment.

To further investigate the long-term effects of levofloxacin exposure on zebrafish embryos, we continued to culture the embryos from the 120 hpf exposure groups in 6-well plates containing 5 mL of embryo culture medium for detoxification treatment. The embryos were fed with paramecium daily, and the water was changed halfway. Mortality was monitored and recorded daily. At 120 hpf, zebrafish enter the mouth-opening stage. If the swim bladder is not developed or inflated properly by this stage, it may impair their swimming and feeding abilities, ultimately leading to death from nutritional deficiencies [54]. Zebrafish larvae at the mouth-opening stage require external nutrients to sustain normal growth and development. Our results showed a significant decrease in the survival rate of zebrafish larvae exposed to 8 mM levofloxacin during the 5–11 dpf period. This likely resulted from a lack of swim bladder inflation, which prevented proper mouth-breathing and feeding, ultimately affecting growth and survival. This outcome is consistent with previous studies on the effects of eugenol exposure on zebrafish swim bladder development, supporting our hypothesis [33]. Furthermore, owing to the small size of zebrafish embryos, they do not fully depend on a functional cardiovascular system during the early stages of development. In the first five days, zebrafish primarily rely on oxygen diffusion from the surrounding water rather than on blood circulation for survival. This allows the larvae to persist and continue developing under relatively normal conditions, even in the presence of cardiovascular defects [35,55]. However, when oxygen diffusion in the water becomes insufficient to sustain life, cardiovascular defects can lead to zebrafish mortality [56]. Therefore, we speculate that exposure to levofloxacin affects zebrafish survival in a dose- and time-dependent manner, potentially linked to cardiac defects. Collectively, these results suggest that even after removal from the exposure environment, the cumulative effects of levofloxacin in zebrafish, whether through swim bladder dysfunction or cardiac toxicity, may have irreversible impacts. In addition, it is equally important to understand the synergistic and antagonistic effects of antibiotic mixtures, as aquatic organisms are often exposed to multiple antibiotics in the aquatic environment at the same time. This study exclusively investigated the effects of single-antibiotic exposure; thus, a key challenge for future environmental toxicity research will be to elucidate the combined effects of co-exposure to multiple antibiotics or other pollutants on aquatic species.

## 5. Conclusions

This study provides a comprehensive evaluation of the toxicological risks posed by levofloxacin exposure during zebrafish development. The findings demonstrate that levofloxacin may represent a significant ecological and environmental threat, particularly to aquatic organisms, due to its dose-dependent developmental toxicity. These results underscore the urgent need for regulating the use and discharge of levofloxacin, alongside the advancement of degradation technologies, to mitigate its detrimental effects on aquatic ecosystems. Further research is imperative to fully understand the long-term impacts of levofloxacin exposure on aquatic species and to devise effective strategies for minimizing its environmental footprint.

## Figures and Tables

**Table 1:** PCR primer sequences.

Primer	Forward Primer (5′→3′)	Reverse Primer (3′→5′)	Length/bp
β-actin	TGGCATCACACCTTCTACAATG	TGGCATCACACCTTCTACAATG	214
nppa	GCTTCCTCTCGGTCTCTGTC	TTGGCAGCAGACGGATGTA	207
myh6	TCTACCTTAGCCAGTGTCAGTT	ACGCAGAGGAACGATGTGA	196
cacna1ab	GAGCCGTGGTGGTGATGAT	GCGTGCGATTGGATGATTACT	280
myl7	TAGCAGCCTCTTGAACTCATCT	TGTCTTCCTCACCCTCTTTGG	130
gata4	AAGTGCGAAGCCGTCCAA	TCTCCACCAGTCCTCCGTTA	154
nkx2.5	GAACATTGGCTAGGTGGTCTC	AAGCAGAGGAAGAGGAGGAAG	124
tbx2b	TGGCTCTCACAATATGGAACCT	TTCACAATTCTCGCTGGATGG	219
tbx5b	TTGGTAAGTTGTGCTGTCTGAA	TCTCGGTCTGAATACACTGTGA	255

## Data Availability

The data that support the findings of this study are available from the corresponding author upon reasonable request.
